# Supplementary material for: Machine Learning Accelerates Crystallization for Structure Determination
Source: Angew Chem Int Ed Engl. 2026 May 4;65(25):e1218503. doi: 10.1002/anie.1218503 (PMC13266958; doi:10.1002/anie.1218503)

checkCIF/PLATON report

Structure factors have been supplied for datablock(s) lcz001

THIS REPORT IS FOR GUIDANCE ONLY. IF USED AS PART OF A REVIEW PROCEDURE FOR PUBLICATION, IT SHOULD NOT REPLACE THE EXPERTISE OF AN EXPERIENCED CRYSTALLOGRAPHIC REFEREE.

No syntax errors found. CIF dictionary Interpreting this report

Datablock: lcz001

```
Bond precision:   C-C = 0.0199 A          Wavelength=1.54184

Cell:              a=8.5029(1)      b=16.2171(2)      c=21.0602(2)
                   alpha=90         beta=90           gamma=90
Temperature:       100 K

                Calculated                    Reported
Volume          2904.04(6)                    2904.04(6)
Space group     P 21 21 21                    P 21 21 21
Hall group      P 2ac 2ab                     P 2ac 2ab
Moiety formula  C21 H7 Ag3 F18 N6 O2          C15 H3 Ag3 F18 N6, C6 H4 O2
Sum formula     C21 H7 Ag3 F18 N6 O2          C21 H7 Ag3 F18 N6 O2
Mr              1040.94                       1040.94
Dx,g cm-3       2.381                         2.381
Z               4                             4
Mu (mm-1)       17.507                        17.507
F000            1976.0                        1976.0
F000'           1986.01
h,k,lmax        10,20,26                      10,19,26
Nref            6246[ 3525]                   5791
Tmin,Tmax       0.115,0.174                   0.221,1.000
Tmin'           0.032

Correction method= # Reported T Limits: Tmin=0.221 Tmax=1.000
AbsCorr = MULTI-SCAN
```

Data completeness= 1.64/0.93 Theta(max)= 78.440

R(reflections)= 0.0538(5493) wR2(reflections)= 0.1527(5791)

S = 1.106 Npar= 452

The following ALERTS were generated. Each ALERT has the format
test-name_ALERT_alert-type_alert-level.
Click on the hyperlinks for more details of the test.

Alert level C

STRVA01_ALERT_4_C Flack test results are ambiguous.
From the CIF: _refine_ls_abs_structure_Flack 0.340
From the CIF: _refine_ls_abs_structure_Flack_su 0.020
PLAT042_ALERT_1_C Calc. and Reported MoietyFormula Strings Differ Please Check
Calc: C21 H7 Ag3 F18 N6 O2
Rep.: C15 H3 Ag3 F18 N6, C6 H4 O2
PLAT090_ALERT_3_C Poor Data / Parameter Ratio (Zmax > 18) .... 7.52 Note
PLAT094_ALERT_2_C Ratio of Maximum / Minimum Residual Density .... 2.09 Report
PLAT342_ALERT_3_C Low Bond Precision on C-C Bonds .... 0.01994 Ang.
PLAT911_ALERT_3_C Missing FCF Refl Between Thmin & STh/L= 0.600 4 Report
1 1 0, 0 11 7, 8 7 11, 0 14 16,
PLAT971_ALERT_2_C Check Calcd Resid. Dens. 0.95Ang From Ag02 1.68 eA-3
PLAT971_ALERT_2_C Check Calcd Resid. Dens. 1.00Ang From N00F 1.66 eA-3
PLAT971_ALERT_2_C Check Calcd Resid. Dens. 1.04Ang From Ag03 1.65 eA-3
PLAT971_ALERT_2_C Check Calcd Resid. Dens. 1.24Ang From N00F 1.64 eA-3
PLAT971_ALERT_2_C Check Calcd Resid. Dens. 1.04Ang From Ag01 1.62 eA-3
PLAT971_ALERT_2_C Check Calcd Resid. Dens. 1.09Ang From Ag02 1.60 eA-3
PLAT971_ALERT_2_C Check Calcd Resid. Dens. 0.92Ang From Ag01 1.56 eA-3
PLAT971_ALERT_2_C Check Calcd Resid. Dens. 1.27Ang From Ag02 1.55 eA-3
PLAT971_ALERT_2_C Check Calcd Resid. Dens. 1.09Ang From Ag01 1.52 eA-3

Alert level G

PLAT003_ALERT_2_G Number of Uiso or U(i,j) Restrained non-H-Atoms 2 Report
PLAT004_ALERT_5_G Polymeric Structure Found with Maximum Dimension 1 Info
PLAT033_ALERT_4_G Flack x Value Deviates > 3.0 * Sigma from Zero . 0.340 Note
PLAT083_ALERT_2_G SHELXL Second Parameter in WGHT Unusually Large 21.96 Why ?
PLAT111_ALERT_2_G ADDSYM Detects New (Pseudo) Centre of Symmetry . 80 %Fit
PLAT113_ALERT_2_G ADDSYM Suggests Possible Pseudo/New Space-group Pnma Check
Check Model Parameter Symmetry for Reflection Data Support
PLAT143_ALERT_4_G s.u. on c - Axis Small or Missing .... 0.00020 Ang.
PLAT186_ALERT_4_G The CIF-Embedded .res File Contains ISOR Records 1 Report
PLAT242_ALERT_2_G Low 'MainMol' Ueq as Compared to Neighbors of C00Z Check
PLAT242_ALERT_2_G Low 'MainMol' Ueq as Compared to Neighbors of C013 Check
PLAT242_ALERT_2_G Low 'MainMol' Ueq as Compared to Neighbors of C017 Check
PLAT242_ALERT_2_G Low 'MainMol' Ueq as Compared to Neighbors of C01A Check
PLAT242_ALERT_2_G Low 'MainMol' Ueq as Compared to Neighbors of C01B Check
PLAT333_ALERT_2_G Large Aver C6-Ring C-C Dist C00Y -C01C . 1.43 Ang.
PLAT335_ALERT_2_G Check Large C6 Ring C-C Range C00Y -C01C 0.17 Ang.
PLAT720_ALERT_4_G Number of Unusual/Non-Standard Labels .... 57 Note
Ag01 Ag02 Ag03 F004 F005 F006 F007 F008
F009 F00A F00B F00C O00D F00E N00F F00G

```
              F00H    F00I    F00J    F00K    F00L    F00M    O00N    F00O
              N00P    N00Q    N00R    N00S    N00T    C00U    C00V    C00W
              C00X    C00Y    C00Z    C010    C011    C012    H012    C013
              C014    H014    C015    C016    C017    C018    H018    C019
              H019    C01A    C01B    C01C    H01C    C01D    H01D    C01E
              H01E
PLAT860_ALERT_3_G Number of Least-Squares Restraints ............         12 Note
PLAT912_ALERT_4_G Missing # of FCF Reflections Above STh/L=  0.600       104 Note
PLAT933_ALERT_2_G Number of HKL-OMIT Records in Embedded .res File         2 Note
                2 18  3,   2 18  4,
PLAT941_ALERT_3_G Average HKL Measurement Multiplicity ..........        3.9 Low
PLAT969_ALERT_5_G The 'Henn et al.' R-Factor-gap value ..........      3.595 Note
              Predicted wR2: Based on SigI**2  4.25 or SHELX Weight 13.80
PLAT978_ALERT_2_G Number C-C Bonds with Positive Residual Density.         0 Info
```

```
   0 ALERT level A = Most likely a serious problem - resolve or explain
   0 ALERT level B = A potentially serious problem, consider carefully
  15 ALERT level C = Check. Ensure it is not caused by an omission or oversight
  22 ALERT level G = General information/check it is not something unexpected

   1 ALERT type 1 CIF construction/syntax error, inconsistent or missing data
  23 ALERT type 2 Indicator that the structure model may be wrong or deficient
   5 ALERT type 3 Indicator that the structure quality may be low
   6 ALERT type 4 Improvement, methodology, query or suggestion
   2 ALERT type 5 Informative message, check
```

Validation response form

Please find below a validation response form (VRF) that can be filled in and pasted into your CIF.

```
# start Validation Reply Form
_vrf_STRVA01_lcz001
;
PROBLEM: Flack test results are ambiguous.
RESPONSE: ...
;
_vrf_PLAT042_lcz001
;
PROBLEM: Calc. and Reported MoietyFormula Strings  Differ     Please Check
RESPONSE: ...
;
_vrf_PLAT090_lcz001
;
PROBLEM: Poor Data / Parameter Ratio (Zmax > 18) ........       7.52 Note
RESPONSE: ...
;
_vrf_PLAT094_lcz001
;
PROBLEM: Ratio of Maximum / Minimum Residual Density ....       2.09 Report
RESPONSE: ...
;
_vrf_PLAT342_lcz001
;
PROBLEM: Low Bond Precision on  C-C Bonds ..............    0.01994 Ang.
RESPONSE: ...
```

```
;
_vrf_PLAT911_lcz001
;
PROBLEM: Missing FCF Refl Between Thmin & STh/L=    0.600          4 Report
RESPONSE: ...
;
_vrf_PLAT971_lcz001
;
PROBLEM: Check Calcd Resid. Dens.  0.95Ang From Ag02            1.68 eA-3
RESPONSE: ...
;
# end Validation Reply Form
```

It is advisable to attempt to resolve as many as possible of the alerts in all categories. Often the minor alerts point to easily fixed oversights, errors and omissions in your CIF or refinement strategy, so attention to these fine details can be worthwhile. It is up to the individual to critically assess their own results and, if necessary, seek expert advice.

PLATON version of 17/09/2025; check.def file version of 17/09/2025

---

duplicate check

No duplication found

Datablock lcz001 - ellipsoid plot

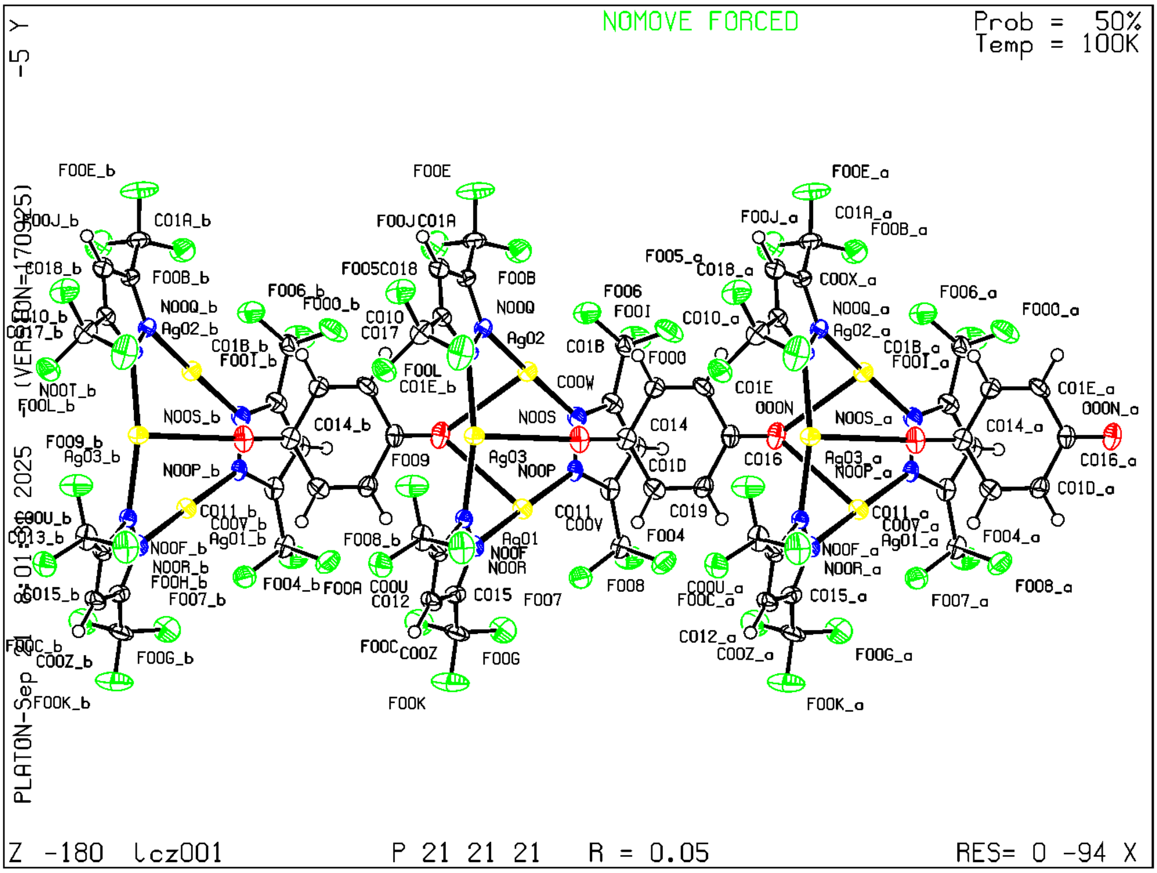

Supplement: Supplementary file 2 — Supporting File 2: anie72462‐sup‐0002‐DataFile.zip. [file ANIE-65-e1218503-s002.zip › supplementary_materials_crystallographic_data submit.zip!/cif_and_checkcif(1-114)/Ag3Pz3_1/Ag3Pz3_1_checkcif.pdf]
